# How does industrial intelligence affect regional innovation efficiency? Evidence from panel data of China’s provinces

**DOI:** 10.1371/journal.pone.0285537

**Published:** 2023-05-05

**Authors:** Kedong Wu, Tianshan Yang, Mengchun Zhu

**Affiliations:** 1 Guangxi University, Nanning, China; 2 Guangxi Vocational University of Agricultural, Nanning, China; Vinnytsia National Technical University, UKRAINE

## Abstract

Innovation efficiency is an important manifestation of regional innovation capacity, and how to improve regional innovation efficiency is crucial to regional development. This study empirically explores the impact of industrial intelligence on regional innovation efficiency and the possible influence of approaches and mechanisms. The empirical results revealed the following. First, the development level of industrial intelligence can positively affect regional innovation efficiency, while beyond a certain level, its role in promoting regional innovation efficiency will weaken, exhibiting an inverted U-shaped effect. Second, compared with the application research by enterprises, industrial intelligence plays a stronger role in promoting the innovation efficiency of basic research by scientific research institutes. Third, human capital situation, financial development level, and industrial structure upgrading are three significant channels for industrial intelligence to promote regional innovation efficiency. Therefore, accelerating the development of industrial intelligence, formulating individualized policies for different innovative entities, and rationally allocating resources related to the development of industrial intelligence are needed to be taken to improve regional innovation.

## Introduction

Innovation is the primary driving force to lead regional development, and innovation efficiency is an important criterion to measure regional innovation capacity. How to improve regional innovation efficiency is a meaningful research topic. According to the theory of knowledge production function, innovation activities can be abstracted as the transformation process from input factors such as R&D personnel, R&D capital, and other R&D resources to output factors such as scientific study, patents, new technologies, and new products. R&D investment and R&D output are only a part of the R&D process, and regions with high R&D investment may not necessarily achieve ideal R&D output. Similarly, regions with high R&D output may also incur a significant wastage of R&D resources. Therefore, this article believes that regional innovation efficiency refers to the efficiency of transforming regional R&D input (R&D personnel, R&D capital, and other R&D resources) into R&D output (patents, new technologies, and new products).

Industry is the pillar of economic development of a geography and has always been valued by many countries. To improve the competitiveness of its industry and seize the opportunity of a new round of industrial revolution, the German government proposed the Industry 4.0 plan to encourage enterprises to carry out intelligent transformation and upgrading of industrial production [1]. To promote the development of digital and intelligent industrial systems in the country, the Japanese government proposed “connected industry” [2]. Further, the Chinese government proposed “Made in China 2025” to promote the intelligent transformation of production equipment in various industries and build a manufacturing powerhouse [3]. Industrial intelligence is based on big data, artificial intelligence, information technology, the Internet of things, and other technologies to achieve functions such as industrial environment self-perception, intelligent optimization and self-decision, precise control, and self-execution [4]. It reconstructs regional industrial production mode, industrial structure, and human capital structure, reduces industrial cost and resource consumption [5, 6], and significantly improves production efficiency and product quality.

Moreover, the influence of industrial intelligentization on regional innovation is also increasing. Some scholars have found that the combination of flexibility and breadth of information technology (IT) enhanced the radicality of innovation and the number of innovations. At the same time, IT integration and deep integration only positively impact the number of innovations [7], and the ability and scale of knowledge accumulation positively impact incremental innovation performance [8]. As the variety of the area increased, the knowledge network structure had an increasing impact on regional innovation [9]. Artificial intelligence affected technological innovation by accelerating knowledge creation and technology spillovers, improving learning and absorptive capacity, and increasing R&D and talent investment [2]. Technological innovation, environmental regulation, and urban green innovation efficiency had significant spatial spillover effects and a U-shaped positive correlation [10, 11].

Regarding the research on industrial intelligence, some scholars believe that industrial intelligence has a positive effect on enterprise innovation. There is a time lag [12], and improving the level of industrial intelligence can effectively promote the positive incentive effect of regional innovation on upgrading industrial structures [13]. Changing the inter-regional industry, the one-way gradient transfer, and the gradient upgrading of regional industrial structure have promoted the agglomeration of business activities in developed cities [14], and the effect of enhancing the output value of enterprises in developed cities is more evident [15]. In addition, industrial intelligence will promote advanced equipment to replace the labor force with junior high and high school education, increase the demand for labor force with high and low education levels, promote the advanced employment structure of the labor force, and positively promote the quality of economic growth [16–18].

To sum up, it can be verified that existing studies have conducted research on innovation activities from the aspects of information technology, artificial intelligence, environmental regulation, and industrialization and confirmed that these factors are essential in influencing innovation or innovation efficiency, which is the impact of industrial intelligence on innovation. These studies provide theoretical and methodological support for the impact of industrial intelligence on innovation and innovation efficiency. However, industrial intelligence is a step-by-step process. What is the relationship between industrial intelligence and innovation efficiency is still unclear. Whether the implementation of industrial intelligence impacts the improvement of innovation efficiency? What are the specific channels of the impact? Whether there exists a heterogeneous impact on the impact of innovation efficiency among different innovation entities?

Further discussions are required. Therefore, this study conducts an in-depth research on the impact of industrial intelligence on innovation efficiency and its channels, clarifies the theoretical mechanism of industrial intelligence affecting innovation efficiency, uses measurement methods to conduct empirical tests, and proposes improvements in the development and reform of industrial intelligence. Policy recommendations on regional innovation efficiency provide a meaningful reference for government decision-making.

### Theoretical mechanisms and hypothesis development

Industrial intelligence empowers enterprises, scientific research institutes, universities, and other organizations to change organizations production methods, production technologies, and management methods, which directly influence the innovation efficiency of enterprises, scientific research institutes, universities, and other innovative subjects. First, industrial intelligence replaces simple and repetitive manual labor with advanced programming, standardization, and automation technologies, realizes scientific management with digitization, informatization, and networking, and provides technical support for data collection, knowledge integration, and scientific management of organizations. Mainly it is the new form of human-computer interaction that can more effectively achieve knowledge retrieval, data processing and scientific management, accelerate the formation of new production methods, new knowledge, and new management models of the organization, and hasten the innovation subject knowledge creation [19].

Furthermore, industrial intelligence can also improve the classification accuracy and management level of the required knowledge, thereby improving the efficiency of regional innovation. Second, the intelligent simulation teaching and training equipment used by universities and scientific research institutes is usually produced by the production line of the enterprise. These industrial intelligent enterprises can produce high-quality intelligent simulation teaching and training equipment, which can be used for universities and research institutes. They can provide basic technical support for simulating the real production environment and application development, enabling universities and research institutes to conduct innovative research orderly and enhance innovation momentum. Good industrial intelligence technology can be applied in enterprises, accelerating the industrial intelligence transformation of enterprises and promoting enterprises to form new products, technologies, and knowledge. And then, industrial intelligence forms knowledge spillover effects in enterprises [20], universities, and research institutes and stimulates the endogenous innovation power of the innovation subject, thereby improving regional innovation efficiency. Finally, people’s energy is limited. The increased energy invested in simple, repetitive, and procedural work will shorten people’s energy to focus on innovation, research, and development, and reduce the innovation efficiency of innovation subjects, while industrial intelligence can liberate scientific researchers to a certain extent. The company’s simple, repetitive and procedural work enables it to devote more energy to innovative R&D activities, thereby increasing the innovation output [21] and ultimately improving the innovation efficiency of each innovation subject. In addition, the phenomenon of employment polarization caused by industrial intelligence has stimulated the demand for high-skilled workers in the region [22]. In an era of rapid upgrading of advanced technology and machinery and equipment, the role of human capital in promoting enterprise innovation is extremely important [23], and is also conducive for increased innovation efficiency. Therefore, this study proposes the first hypothesis.

**Hypothesis 1:** Under other conditions being equal, developing industrial intelligence can promote the improvement of regional innovation efficiency.

The development of industrial intelligence accelerates the formation of innovative entities in information acquisition, knowledge creation, and scientific management, and the demand for labor is more inclined to high-skilled labor, which accelerates the transformation of talents to high-skilled innovation and improves the efficiency of R&D personnel in the innovation subject to obtain the required innovation information. It advances technology and cutting-edge knowledge and improves the ability of technology transformation and application, thereby accelerating the optimization and accumulation of human capital, making human capital advanced. The process of industrialization has gained a boost [24]. It is precisely the industrial intelligence that changes the labor structure, improves the level of human capital, continuously stimulates the innovation vitality of innovation entities, promotes a substantial increase in innovation output, and ultimately improves regional innovation efficiency. Therefore, this study proposes a second hypothesis.

**Hypothesis 2:** Industrial intelligence can optimize human capital, affecting regional innovation efficiency.

Innovation activities have high sunk costs and are considered typical high-investment and high-risk activities. It can be seen that orderly operation of each link of innovation activities requires a large amount of capital investment, and the financial sector is required to provide strong support for innovative research and development experiments, production technology improvement, technology promotion, and promote the innovation output of innovation entities. Industrial intelligence provides a convenient innovation infrastructure for innovators, enabling labor to be replaced by cheaper capital, reducing redundant labor, and improving the output of innovators. At the same time, industrial intelligence enables innovators to search for more information. The convenience of information acquisition has increased.

Moreover, the level of knowledge spillover has improved. The uncertainty in the R&D process of innovative entities has been dramatically reduced. The credibility of innovative entities has been improved, and the willingness of the financial sector to invest in innovation has been activated. The financing speed has been improved, and the speed of capital flow has accelerated. Therefore, the efficiency of capital allocation has been improved. It can provide the possibility to expand the scale of financial supply and demand. The improvement of capital allocation efficiency and the expansion of the financial development scale has a positive impact on the innovation efficiency of each innovation subject. Therefore, this study proposes a third hypothesis.

**Hypothesis 3:** Industrial intelligence promotes financial development, affecting regional innovation efficiency.

Industrial intelligence improves the use efficiency of production factors. It optimizes the allocation level of production factors, thus giving birth to new models, new products, and new departments and promoting the transformation and upgrading of traditional industries to medium and high-end industries. Post industrial upgrades, the production sector will put forward higher requirements for the innovation and R&D department. They will force the main body of innovation to continue to carry out further technological innovation rather than merely being satisfied with the current level of innovation. And then will promote the continuous and rapid development of innovation activities. Ultimately, the efficiency of innovation will be improved. Therefore, this study proposes a fourth hypothesis.

**Hypothesis 4:** Industrial intelligence promotes industrial optimization and upgrading, which in turn affects regional innovation efficiency.

### Econometric model, variables, and data description

In this section, we set up econometric models to examine the theoretical propositions regarding the effect of industrial intelligence on innovation efficiency by using panel data from China’s provinces.

### Econometric model

According to the previous theoretical analysis and the proposed research hypotheses, the following model is set to estimate the impact of industrial intelligence development on China’s innovation efficiency:

$$te_{it}=\alpha_{0}+\alpha_{1}INT_{it}+\sum_{j}\alpha_{j}X_{it}^{j}+\mu_{i}+\nu_{t}+\varepsilon_{it}$$ (1)

where the subscripts *i* and *t* respectively represent a particular province and year; *te* is regional innovation efficiency; *INT* is the development level of industrial intelligence; *X*^*j*^ refers to other control variables that influence regional innovation efficiency; *α*_0_ is the constant term; *α*_1_ and *α*_*j*_ are coefficients, which reflect the impact of artificial intelligence and control variables on innovation efficiency; *μ* and *ν* control for individual factors and time factors, respectively; *ε* is a random error terms. Further, a mediation effect model is constructed to estimate the effect path of industrial intelligence on innovation efficiency:

$$Med_{it}=\beta_{0}+\beta_{1}INT_{it}+\sum_{j}\beta_{j}X_{it}^{j}+\mu_{i}^{Med}+\nu_{t}^{Med}+\varepsilon_{it}^{Med}$$ (2)

$$te_{it}=\gamma_{0}+\gamma_{1}INT_{it}+\gamma_{2}Med_{it}+\sum_{j}\gamma_{j}X_{it}^{j}+\mu_{i}^{l}+\nu_{t}^{l}+\varepsilon_{it}^{l}$$ (3)

where *Med* is the mediator variable; *β*_0_, *γ*_0_ are the constant terms; *β*_1_, *γ*_1_, *γ*_2_, *β*_*j*_, and *γ*_*j*_ are coefficients; *μ*^*Med*^, *μ*^*l*^, *ν*^*Med*^, and *ν*^*l*^ control for individual and time factors, respectively; *ε*^*Med*^ and *ε*^*l*^ are random error terms. The other variables are as described above.

### Description of data and variables

This study uses the 2010–2019 Chinese provincial panel data (excluding Tibet data) for empirical testing.

The data are mainly sourced from the 2011–2020 China Statistical Yearbook, the 2011–2020 China Financial Statistical Yearbook, and the CSMAR database over the years. Descriptive statistics for each variable are shown in Table 1, and the variables in the empirical testing are set as follows:

- Dependent Variables. Innovation efficiency is the explained variable of this study. Referring to the existing literature, based on the input output perspective, SFA(Stochastic Frontier Approach) method is selected to calculate the innovation efficiency of each province in China. Specifically, innovation output is represented by the number of patent applications authorized; the full-time equivalent of R&D personnel represents labor input; and the internal expenditure of R&D funds represents capital input. For the selection of the SFA model form, we calculate the Cobb-Douglas production function model and the transcendental logarithmic stochastic frontier model and use the generalized likelihood rate technique to test the model’s suitability. The results show that exceeding the logarithmic stochastic frontier technology is more suitable for measuring regional innovation efficiency and the innovation efficiency of the three major innovation entities.
- Core independent variables. Industrial intelligence development level measurement index (*INT*). Based on the existing research results, this study builds an index system based on three aspects: industrial intelligent infrastructure construction, industrial intelligent production application, and industrial intelligent competitiveness and efficiency (Sun, Hou, 2019).
- Control Variables. Referring to the existing literature, this study controls the following variables: the level of urbanization (urb), which is measured by the proportion of urban population to the total population at the end of the year. Trade openness (tra), is measured by the ratio of total imports and exports to GDP. Government intervention (gov) is represented by the proportion of fiscal expenditure in each region to GDP. Intellectual property protection (ipr), is measured by the proportion of technology market transactions in GDP. R&D investment intensity (rd) is measured by the ratio of R&D investment to GDP. Marketization level (mar), expressed as the proportion of non-state-owned enterprise employees to all employees.
- Mediating Variables. Human capital (hc), expressed by the proportion of employees with a college degree or above in the total number of jobs in each region; financial development (fin), measured by the ratio of the year-end deposit balance of financial institutions in each region to GDP; industrial upgrading (stu) is measured by the ratio of the output value of the tertiary to the secondary industry in each region.

**Table 1:** Descriptive statistics.

Variable	Mean	St. Dev	Min	Max
int	14.561	8.694	2.150	57.740
te	0.324	0.182	0.053	1.000
hc	0.130	0.089	0.030	0.559
fin	1.602	0.706	0.108	5.587
stu	0.934	0.507	0.500	4.035
urb	0.524	0.139	0.275	0.896
tra	0.329	0.394	0.009	1.721
gov	0.215	0.094	0.084	0.627
ipr	0.010	0.022	0.000	0.150
rd	0.014	0.011	0.002	0.060
mar	0.696	0.100	0.440	0.886

## Regression results

### Baseline regression analyses

This study first examines the direct impact of industrial intelligence on the innovation efficiency of three major innovation subjects by using the overall sample. To reduce the possible influence of regional and time factors on model estimation, all models control the regional and time factors and select the fixed-effect model for estimation based on the Hausman test. The baseline regression results are shown in Table 2. In the table, Model 1 shows that the improvement of the development level of industrial intelligence has a positive impact on the innovation efficiency of all provinces in the country, and the coefficient is significant at the 1% level, confirming Hypothesis 1.

**Table 2:** Baseline regression results.

	Model 1	Model 2	Model 3	Model 4	Model 5	Model 6	Model 7
INT	0.000381***	0.422***	0.000315***	0.0312***	0.000341***	0.00217*	0.000366***
	(0.0000642)	(0.0818)	(0.0000661)	(0.00564)	(0.0000675)	(0.00114)	(0.0000643)
urb	0.0390***	25.40***	0.0350***	-1.969***	0.0414***	0.0719	0.0385***
	(0.00541)	(6.896)	(0.00545)	(0.475)	(0.00556)	(0.0963)	(0.00539)
tra	-0.00358***	-0.903	-0.00343***	-0.0694	-0.00349***	-0.0501***	-0.00324***
	(0.00104)	(1.323)	(0.00102)	(0.0912)	(0.00103)	(0.0185)	(0.00105)
gov	0.0118***	11.67**	0.00999***	2.660***	0.00847**	0.0858	0.0112***
	(0.00355)	(4.523)	(0.00353)	(0.312)	(0.00399)	(0.0631)	(0.00354)
ipr	0.0531**	112.1***	0.0355*	-0.608	0.0538**	-0.107	0.0538**
	(0.0211)	(26.87)	(0.0214)	(1.852)	(0.0210)	(0.375)	(0.0210)
rd	0.0319	373.8***	-0.0266	20.80***	0.00571	1.812	0.0196
	(0.0637)	(81.17)	(0.0650)	(5.594)	(0.0650)	(1.133)	(0.0636)
mar	0.0220***	13.35***	0.0199***	-0.230	0.0223***	0.0701	0.0215***
	(0.00269)	(3.426)	(0.00271)	(0.236)	(0.00268)	(0.0478)	(0.00268)
hc			0.000156***				
			(0.0000475)				
fin					0.00126*		
					(0.000699)		
stu							0.00677*
							(0.00345)
_cons	0.664***	-24.24***	0.667***	1.504***	0.662***	0.267***	0.662***
	(0.00216)	(2.755)	(0.00241)	(0.190)	(0.00240)	(0.0385)	(0.00234)
Province FE	yes	yes	yes	yes	yes	yes	yes
Year FE	yes	yes	yes	yes	yes	yes	yes
R-squared	0.880	0.826	0.885	0.631	0.881	0.376	0.882

Notes: Robust standard errors are in parentheses

*p<0.1

**p<0.05

***p<0.01

Second, this study further examines the indirect impact of the development of industrial intelligence on the innovation efficiency of three major innovation entities. We mainly test from three aspects: first, we test the indirect effect of human capital. The estimated results are shown in Model 2 and Model 3 in Table 2. Model 2 implies that the development of industrial intelligence positively influences human capital, and the coefficient is significant at the 1% level. Model 3 indicates that human capital positively influences innovation efficiency, and the coefficient is significant at the 1% level, indicating that human capital is an important path for industrial intelligence to indirectly improve innovation efficiency. The indirect effect accounts for 17.3% of the total effect, which confirms Hypothesis 2. Second, the indirect effect of financial development is tested. The estimated results are shown in Model 4 and 5 in Table 2. Model 4 shows that the development of industrial intelligence positively influences financial development, and the coefficient is significant at the 1% level. Model 5 shows that financial development has a positive impact on innovation efficiency, and the coefficient is significant at the 10% level, indicating that financial development is another important path for industrial intelligence to indirectly improve innovation efficiency, and the indirect effect accounts for 10.3% of the total effect, confirming Hypothesis 3.

Third, the indirect effect of industrial upgrading is tested, and the estimated results are shown in Model 6 and 7 in Table 2. Model 6 indicates that the development of industrial intelligence positively influences industrial upgrading, and the coefficient is significant at the 10% level. Model 7 shows that industrial upgrading positively impacts innovation efficiency, and the coefficient is significant at the 10% level, which indicates that industrial upgrading is another vital path for industrial intelligence to indirectly improve innovation efficiency. The indirect effect accounts for 3.9% of the total effect, confirming Hypothesis 4.

It is worth noting that the R^2^ of Model 6 in Table 2 is significantly smaller than that of other models, and the significance level of the impact coefficient of industrial intelligence on industrial upgrading in Model 6 is relatively high. This indicates that compared to other models, the fitting effect of Model 6 is poor, which also means that the results of industrial intelligence promoting industrial upgrading must be carefully used.

### Regression analysis of different innovation subjects

Considering the differences in the innovation capabilities of different innovation entities, it is necessary to deeply analyze the impact of industrial intelligence on the innovation efficiency of different innovation entities. Table 3 presents the effect of industrial intelligence on enterprise innovation efficiency. It can be seen from this that the effect coefficient of industrial intelligence on enterprise innovation efficiency is positive and significant at the 1% level. Human capital, financial development, and industrial upgrading are the three paths for industrial intelligence to improve the innovation efficiency of enterprises. Table 4 shows the effect of industrial intelligence on the innovation efficiency of colleges and universities. It can be seen that the effect coefficient of industrial intelligence on the innovation efficiency of colleges and universities is positive, and it is significant at the 1% level. Human capital, financial development, and industrial upgrading are the three paths for industrial intelligence to improve the innovation efficiency of colleges and universities. Table 5 shows the effect of industrial intelligence on the innovation efficiency of scientific research institutes. It can be seen that the effect coefficient of industrial intelligence on the innovation efficiency of scientific research institutes is positive and significant at the 1% level. Human capital, financial development, and industrial upgrading are the three paths for industrial intelligence to promote the innovation efficiency of scientific research institutes.

**Table 3:** Regression results for firms of innovation efficiency.

	Model 1	Model 2	Model 3	Model 4	Model 5	Model 6	Model 7
INT	0.00683***	0.422***	0.00590***	0.0312***	0.00638***	0.00217*	0.00654***
	(0.000770)	(0.0818)	(0.000787)	(0.00564)	(0.000810)	(0.00114)	(0.000762)
urb	0.790***	25.40***	0.734***	-1.969***	0.818***	0.0719	0.780***
	(0.0649)	(6.896)	(0.0649)	(0.475)	(0.0667)	(0.0963)	(0.0638)
tra	-0.0515***	-0.903	-0.0496***	-0.0694	-0.0505***	-0.0501***	-0.0449***
	(0.0124)	(1.323)	(0.0121)	(0.0912)	(0.0124)	(0.0185)	(0.0124)
gov	0.263***	11.67**	0.237***	2.660***	0.224***	0.0858	0.251***
	(0.0426)	(4.523)	(0.0420)	(0.312)	(0.0479)	(0.0631)	(0.0420)
ipr	-0.396	112.1***	-0.641**	-0.608	-0.387	-0.107	-0.382
	(0.253)	(26.87)	(0.254)	(1.852)	(0.252)	(0.375)	(0.248)
rd	0.660	373.8***	-0.156	20.80***	0.360	1.812	0.421
	(0.764)	(81.17)	(0.774)	(5.594)	(0.781)	(1.133)	(0.754)
mar	0.241***	13.35***	0.211***	-0.230	0.244***	0.0701	0.231***
	(0.0322)	(3.426)	(0.0323)	(0.236)	(0.0322)	(0.0478)	(0.0318)
hc			0.00218***				
			(0.000565)				
fin					0.0144*		
					(0.00839)		
stu							0.132***
							(0.0408)
_cons	-0.301***	-24.24***	-0.248***	1.504***	-0.323***	0.267***	-0.336***
	(0.0259)	(2.755)	(0.0287)	(0.190)	(0.0287)	(0.0385)	(0.0277)
Province FE	yes	yes	yes	yes	yes	yes	yes
Year FE	yes	yes	yes	yes	yes	yes	yes
R-squared	0.937	0.826	0.941	0.631	0.938	0.376	0.940

Notes: Robust standard errors are in parentheses. *p<0.1; **p<0.05; ***p<0.01

**Table 4:** Regression results for colleges and universities of innovation efficiency.

	Model 1	Model 2	Model 3	Model 4	Model 5	Model 6	Model 7
INT	0.00748***	0.422***	0.00621***	0.0312***	0.00669***	0.00217*	0.00679***
	(0.000692)	(0.0818)	(0.000680)	(0.00564)	(0.000716)	(0.00114)	(0.000593)
urb	0.831***	25.40***	0.754***	-1.969***	0.881***	0.0719	0.808***
	(0.0583)	(6.896)	(0.0560)	(0.475)	(0.0590)	(0.0963)	(0.0497)
tra	-0.0252**	-0.903	-0.0224**	-0.0694	-0.0234**	-0.0501***	-0.00914
	(0.0112)	(1.323)	(0.0105)	(0.0912)	(0.0110)	(0.0185)	(0.00965)
gov	0.174***	11.67**	0.139***	2.660***	0.107**	0.0858	0.147***
	(0.0382)	(4.523)	(0.0362)	(0.312)	(0.0424)	(0.0631)	(0.0327)
ipr	-0.713***	112.1***	-1.051***	-0.608	-0.698***	-0.107	-0.679***
	(0.227)	(26.87)	(0.220)	(1.852)	(0.223)	(0.375)	(0.193)
rd	2.553***	373.8***	1.426**	20.80***	2.025***	1.812	1.973***
	(0.686)	(81.17)	(0.668)	(5.594)	(0.690)	(1.133)	(0.587)
mar	0.259***	13.35***	0.219***	-0.230	0.265***	0.0701	0.237***
	(0.0290)	(3.426)	(0.0279)	(0.236)	(0.0285)	(0.0478)	(0.0247)
hc			0.00301***				
			(0.000488)				
fin					0.0254***		
					(0.00742)		
stu							0.320***
							(0.0318)
_cons	-0.530***	-24.24***	-0.457***	1.504***	-0.569***	0.267***	-0.616***
	(0.0233)	(2.755)	(0.0248)	(0.190)	(0.0254)	(0.0385)	(0.0216)
Province FE	yes	yes	yes	yes	yes	yes	yes
Year FE	yes	yes	yes	yes	yes	yes	yes
R-squared	0.953	0.826	0.959	0.631	0.955	0.376	0.966

Notes: Robust standard errors are in parentheses. *p<0.1; **p<0.05; ***p<0.01

**Table 5:** Regression results for scientific research institutes of innovation efficiency.

	Model 1	Model 2	Model 3	Model 4	Model 5	Model 6	Model 7
INT	0.00994***	0.422***	0.00832***	0.0312***	0.00942***	0.00217*	0.00936***
	(0.00083)	(0.0818)	(0.00080)	(0.00564)	(0.00087)	(0.00114)	(0.000775)
urb	0.867***	25.40***	0.769***	-1.969***	0.900***	0.0719	0.848***
	(0.0697)	(6.896)	(0.0663)	(0.475)	(0.0716)	(0.0963)	(0.0649)
tra	-0.0272**	-0.903	-0.0237*	-0.0694	-0.0260*	-0.0501***	-0.0138
	(0.0134)	(1.323)	(0.0124)	(0.0912)	(0.0133)	(0.0185)	(0.0126)
gov	0.371***	11.67**	0.326***	2.660***	0.326***	0.0858	0.348***
	(0.0457)	(4.523)	(0.0429)	(0.312)	(0.0514)	(0.0631)	(0.0427)
ipr	-0.783***	112.1***	-1.213***	-0.608	-0.773***	-0.107	-0.755***
	(0.272)	(26.87)	(0.260)	(1.852)	(0.270)	(0.375)	(0.253)
rd	2.654***	373.8***	1.222	20.80***	2.308***	1.812	2.169***
	(0.820)	(81.17)	(0.791)	(5.594)	(0.838)	(1.133)	(0.767)
mar	0.272***	13.35***	0.221***	-0.230	0.276***	0.0701	0.253***
	(0.0346)	(3.426)	(0.0330)	(0.236)	(0.0345)	(0.0478)	(0.0324)
hc			0.00383***				
			(0.000578)				
fin					0.0167*		
					(0.00900)		
stu							0.268***
							(0.0416)
_cons	-0.535***	-24.24***	-0.442***	1.504***	-0.560***	0.267***	-0.606***
	(0.0278)	(2.755)	(0.0294)	(0.190)	(0.0308)	(0.0385)	(0.0282)
Province FE	yes	yes	yes	yes	yes	yes	yes
Year FE	yes	yes	yes	yes	yes	yes	yes
R-squared	0.955	0.826	0.961	0.631	0.956	0.376	0.961

Notes: Robust standard errors are in parentheses. *p<0.1; **p<0.05; ***p<0.01

From the perspective of the effect of industrial intelligence on the innovation efficiency of enterprises, universities, and scientific research institutes, it shows the characteristics of “the largest scientific research institute, followed by universities, and the smallest enterprise.” This implies that compared with applied research represented by enterprises, industrial intelligence plays a stronger role in promoting the innovation efficiency of basic research represented by scientific research institutes.

### Regression analysis of the influence effect of different industrial intelligence levels

To further study the impact of different industrial intelligence levels on innovation efficiency, quantile regression is used for estimation. Table 6 shows that with the improvement of the development level of industrial intelligence, the impact of industrial intelligence on innovation efficiency presents an inverted “U” shape. That is, when the development level of industrial intelligence is low, the positive effect of industrial intelligence on innovation efficiency gradually increases. With the increase in the development level of industrial intelligence, the positive influence of industrial intelligence on innovation efficiency gradually decreases.

**Table 6:** Quantile regression results of the effect of industrial intelligence level.

	QR_10	QR_20	QR_30	QR_40	QR_50	QR_60	QR_70	QR_80	QR_90
INT	0.00875***	0.00961***	0.00984***	0.0122***	0.0134***	0.0160***	0.0160***	0.0132***	0.0131***
	(0.000351)	(0.000664)	(0.000988)	(0.00165)	(0.00202)	(0.00228)	(0.00215)	(0.00199)	(0.00153)
fin	0.0131***	0.0207**	0.00844	0.00835	0.0150	0.0455	0.0595**	0.120***	0.151***
	(0.00491)	(0.00930)	(0.0138)	(0.0231)	(0.0284)	(0.0319)	(0.0302)	(0.0279)	(0.0214)
urb	-0.537***	-0.556***	-0.629***	-0.722***	-0.713***	-0.507***	-0.557***	-0.572***	-0.613***
	(0.0187)	(0.0353)	(0.0525)	(0.0877)	(0.108)	(0.121)	(0.115)	(0.106)	(0.0813)
tra	0.0997***	0.0871***	0.118***	0.132***	0.134***	0.116***	0.103***	-0.00729	-0.0217
	(0.00624)	(0.0118)	(0.0176)	(0.0293)	(0.0360)	(0.0405)	(0.0383)	(0.0354)	(0.0272)
gov	0.268***	0.250***	0.233***	0.282***	0.316**	0.235	0.339**	-0.0920	-0.289***
	(0.0224)	(0.0424)	(0.0630)	(0.105)	(0.129)	(0.145)	(0.137)	(0.127)	(0.0975)
ipr	-2.142***	-2.217***	-2.076***	-2.333***	-2.927***	-2.840***	-3.700***	-4.278***	-4.991***
	(0.142)	(0.269)	(0.401)	(0.669)	(0.821)	(0.923)	(0.874)	(0.807)	(0.620)
rd	-3.217***	-3.644***	-2.996***	-3.468**	-3.408	-9.468***	-7.253***	-6.131***	-5.680***
	(0.365)	(0.691)	(1.027)	(1.714)	(2.105)	(2.367)	(2.240)	(2.070)	(1.589)
mar	0.121***	0.120***	0.0576	0.0465	-0.0142	-0.0505	-0.0713	-0.103	-0.261***
	(0.0177)	(0.0336)	(0.0500)	(0.0834)	(0.102)	(0.115)	(0.109)	(0.101)	(0.0773)
_cons	0.653***	0.664***	0.759***	0.789***	0.813***	0.788***	0.788***	0.918***	1.077***
	(0.0161)	(0.0305)	(0.0454)	(0.0757)	(0.0930)	(0.105)	(0.0990)	(0.0914)	(0.0702)
Province FE	yes	yes	yes	yes	yes	yes	yes	yes	yes
Year FE	yes	yes	yes	yes	yes	yes	yes	yes	yes

Notes: Robust standard errors are in parentheses. *p<0.1; **p<0.05; ***p<0.01

## Robustness checks

To further enhance the robustness of the research conclusions and deal with endogeneity, the model was re-estimated by taking the one lag period of the core explanatory variable as the instrumental variable of the current period, and the results are shown in Table 7. It can be observed that the estimated results are consistent with the benchmark regression results, and the coefficient signs and significances of the core explanatory variables and mediator variables are the same as the previous ones, which shows that the research hypotheses of this study are still valid after considering the endogeneity problem.

**Table 7:** Instrumental variable estimation results.

	Model 1	Model 2	Model 3	Model 4	Model 5	Model 6	Model 7
INT	0.000525***	0.742***	0.000449***	0.0371***	0.000510***	0.00243	0.000507***
	(0.0000957)	(0.131)	(0.000104)	(0.00891)	(0.0000957)	(0.00174)	(0.0000958)
urb	0.0314***	11.81	0.0301***	-1.933***	0.0306***	0.155	0.0302***
	(0.00667)	(9.170)	(0.00654)	(0.622)	(0.00658)	(0.122)	(0.00658)
tra	-0.00265**	0.0449	-0.00266**	-0.238**	-0.00257**	-0.0669***	-0.00213*
	(0.00123)	(1.684)	(0.00120)	(0.114)	(0.00121)	(0.0223)	(0.00123)
gov	0.0137***	13.41**	0.0124***	2.758***	0.0120***	0.131*	0.0127***
	(0.00380)	(5.217)	(0.00378)	(0.354)	(0.00383)	(0.0692)	(0.00378)
ipr	0.0261	59.37*	0.0200	-2.146	0.0312	-0.406	0.0293
	(0.0236)	(32.49)	(0.0232)	(2.202)	(0.0237)	(0.431)	(0.0235)
rd	-0.0375	300.7***	-0.0685	15.64**	-0.0508	0.872	-0.0442
	(0.0681)	(93.61)	(0.0680)	(6.347)	(0.0674)	(1.241)	(0.0673)
mar	0.0217***	15.95***	0.0201***	-0.113	0.0219***	0.0989**	0.0210***
	(0.00271)	(3.725)	(0.00277)	(0.253)	(0.00268)	(0.0494)	(0.00270)
high			0.000103**				
			(0.0000501)				
fin					0.00128**		
					(0.000588)		
x8							0.00775**
							(0.00359)
_cons	0.666***	-22.97***	0.668***	1.425***	0.667***	0.209***	0.665***
	(0.00263)	(3.610)	(0.00276)	(0.245)	(0.00259)	(0.0479)	(0.00275)
Province FE	yes	yes	yes	yes	yes	yes	yes
Year FE	yes	yes	yes	yes	yes	yes	yes

Notes: Robust standard errors are in parentheses. *p<0.1; **p<0.05; ***p<0.01

## Conclusions and policy recommendations

This study theoretically constructs the path that industrial intelligence influences regional innovation efficiency and empirically tests the research hypothesis based on China’s provincial panel data. It is found that: first, the improvement of the development level of industrial intelligence can significantly promote regional innovation efficiency. Second, industrial intelligence can promote innovation efficiency through three paths: human capital, financial development, and industrial upgrading. Third, the effect of industrial intelligence on the innovation efficiency of enterprises, universities, and research institutes shows heterogeneity. Compared with applied research represented by enterprises, industrial intelligence promotes the development of basic research represented by scientific research institutes. The role of innovation efficiency improvement is stronger. Fourth, with the improvement of the development level of industrial intelligence, the effect of industrial intelligence on innovation efficiency presents an inverted “U” shape.

In this regard, we present the following recommendations. First, accelerate the development of industrial intelligence and improve the level of industrial intelligence in various regions of China. Specifically, strengthen the construction of industrial intelligent infrastructure, while taking into account balanced development among various regions in China. In line with the development trend of industrial intelligence and digitalization, actively develop emerging business formats with industrial intelligence as the core, improve the intelligence and industrial level of integration with the service industry, to achieve the integration and innovation of various industries and intelligent development.

Second, formulate targeted innovation policies based on the respective characteristics of different innovation entities. Encourage scientific research institutes and universities to actively carry out industry-university-research cooperation with enterprises based on theoretical innovation and original innovation to accelerate the transformation of innovation achievements; guide enterprises to keep up with the trend of intelligent development, build intelligent innovation development ideas, and cooperate with scientific research innovation. Unit cooperation promotion, module outsourcing, and other methods are used to address the innovation bottleneck encountered.

Finally, rationally allocate resources related to the development of industrial intelligence. As an emerging technology, industrial intelligence must be reasonably equipped with corresponding supporting resources to fully play the role of industrial intelligence in promoting innovation efficiency. Therefore, it is necessary to continuously optimize the human capital structure, support the financial business to tilt towards the field of industrial intelligence, adjust and upgrade the industrial structure in a timely manner, realize the rational allocation and dynamic adjustment of various elements, and fully release the innovative effect of industrial intelligence.
